# A Comparative Genetic Analysis of *Phoenix atlantica* in Cape Verde

**DOI:** 10.3390/plants13162209

**Published:** 2024-08-09

**Authors:** Sonia Sarmiento Cabello, Priscila Rodríguez-Rodríguez, Guacimara Arbelo Ramírez, Agustín Naranjo-Cigala, Leticia Curbelo, Maria de Monte da Graca Gomes, Juliana Brito, Frédérique Aberlenc, Salwa Zehdi-Azouzi, Pedro A. Sosa

**Affiliations:** 1 Instituto Universitario de Estudios Ambientales y Recursos Naturales (IUNAT), Universidad de Las Palmas de Gran Canaria, Campus Universitario de Tafira, 35017 Las Palmas de Gran Canaria, Spain; 2 Direção Geral Da Agricultura Silvicultura e Pecuaria e Delegação do Ministerio da Agricultura e Ambiente do Sal e da Boavista, Praia, Cape Verde; 3 Plant Diversity, Adaptation and Development, Université de Montpellier, Institut de Recherche pour Développement, Centre de Coopération Internationale en Recherche Agronomique pour le Développement, 911 Av. Agropolis, BP 64501, 34394 Montpellier CEDEX 5, France; 4 Laboratoire de Génétique Moléculaire, Faculté des Sciences de Tunis, Immunologie et Biotechnologie (LR99ES12), Université de Tunis El Manar, Campus Universitaire Farhat Hached, Tunis 1068, Tunisia

**Keywords:** *Araceae*, plant conservation, conservation genetics, population genetics, microsatellites, minisatellites, oceanic islands

## Abstract

**Simple Summary:**

This study genetically characterizes the Cape Verde palm tree, *Phoenix atlantica*, exploring its genetic differentiation and recent divergence from its relative, *Phoenix dactylifera*, while also examining its significance for conservation efforts and enhancing our understanding of the historical trajectories of African palm trees.

**Abstract:**

The Cape Verde palm tree, *Phoenix atlantica*, holds significant ecological and cultural importance within the Cape Verde archipelago. However, its genetic distinctiveness has been questioned due to its close relationship and morphological similarity to the date palm (*Phoenix dactylifera*). In this study, we used an expanded sample set, 18 simple sequence repeat (SSR) markers, and a plastid minisatellite to characterize *P. atlantica* in Cape Verde and investigate its relationship with other *Phoenix* species. Our findings identify genetic markers that differentiate the *P. atlantica* genetic pool, including a unique fixed allele. We also provide evidence of the recent divergence of *P. atlantica* from Northern African date palm populations, suggesting a relatively recent colonization of Cape Verde by palm trees. Additionally, we characterized the genetic composition of palm tree populations across three Cape Verde islands, concluding that wild samples from certain populations in Boavista and Sal are best suited for establishing a seed and/or germplasm bank for replantation efforts, representing a crucial step for the conservation of Cape Verde’s natural heritage. Overall, our results enhance the understanding of the historical trajectories and genetic characterization of palm trees in Africa, offering valuable insights for conservation strategies.

## 1. Introduction

The *Phoenix* genus, composed of 14 species [1], holds considerable significance owing to its multifaceted contributions, encompassing edible fruits, an ornamental appearance, and profound religious importance [2]. The most notable representative of this genus is the widely cultivated date palm (*Phoenix dactylifera*), spanning from North Africa to West Asia. This species plays a pivotal role in arid regions, particularly in North Africa and the Middle East, producing sugar-rich, nutritious dates that have been integral to the local diet for centuries [3,4], which reached 9.6 million tons of production in 2021 [5].

Despite its economic and cultural importance, the *Phoenix* genus faces challenges arising from interspecific hybridization, resulting in difficulties in distinguishing distinct species within the genus and raising concerns for conservation efforts [2]. Molecular techniques have thus become crucial in its genetic characterization and have been key in offering insights into species boundaries and their genetic relationships [2,6,7,8]). Despite these advancements, the characterization of certain species, such as the Cape Verde palm tree (*Phoenix atlantica*), remains a challenge.

The Cape Verde palm tree, *P. atlantica*, is the only endemic species of the *Phoenix* genus in Cape Verde. In contrast, *P. dactylifera* and *P. canariensis* have been primarily introduced in the archipelago artificially by humans [9]. *P. atlantica* exhibits distinct morphological features compared to *P. canariensis*, but its differences from *P. dactylifera* are more subtle [7,10,11]. Initially, genetic studies identified *P. atlantica* as a distinct species within the *Phoenix* genus, identifying 69 private alleles in at least 6 distinct loci [11]. Nevertheless, more recent whole-genome and chloroplast genetic analyses indicate that *P. atlantica* belongs to the same genetic cluster as *P. dactylifera*, suggesting that *P. atlantica* might be a feral form of the date palm [12,13,14].

Cape Verde, situated in the Atlantic Ocean, has recently garnered attention for its remarkable diversity of endemic species [15,16]. Notably, *P. atlantica* is one of only six native trees and one of just three endemic trees in the archipelago [17,18], prompting a critical examination of its genetic composition and differentiation from its close relative, *P. dactylifera*. Beyond its biological significance, this palm tree serves as a valuable resource for the local population, providing building materials, sustenance for livestock, and consumed and traded fruits across the islands [9]. Additionally, as Cape Verde undergoes an economic surge propelled by the expansion of tourism [19], the imperative for conservation becomes paramount. Thus, understanding and safeguarding the genetic heritage of *P. atlantica* represent a key step in both the development of and nature conservation in Cape Verde.

In this context, the present study aims to genetically characterize *P. atlantica* populations in Cape Verde and delve into their relationships with other *Phoenix* species. Specifically, our goal is to identify and compare genetic markers, assess allele frequencies, and examine patterns of genetic variation. For this purpose, we use 18 biparentally inherited nuclear microsatellites and one maternally inherited chloroplast minisatellite previously used for *P. dactylifera* [20] and *P. canariensis* [21] and for comparing different species in the genus [6,22,23]. Unlike previous studies that relied on a limited number of samples, our approach involves a thorough genetic analysis using an increased sample size. Through these efforts, we aim to enhance our insights into the genetic diversity and structure of *P. atlantica*.

## 2. Materials and Methods

### 2.1. Study Area and Sampling

The Republic of Cape Verde, located in the Atlantic Ocean, consists of nine inhabited volcanic islands, one uninhabited volcanic island, and several islets. Positioned approximately 500 km off the westernmost point of the African mainland and 1500 km south of the Canary Islands, the archipelago showcases mostly rugged and mountainous terrain. Notably, three islands—Sal, Boavista, and Maio—contrastingly feature flat desert landscapes adorned with sandy beaches. Precipitation is scarce and highly irregular across the archipelago [9].

**Figure 1:**
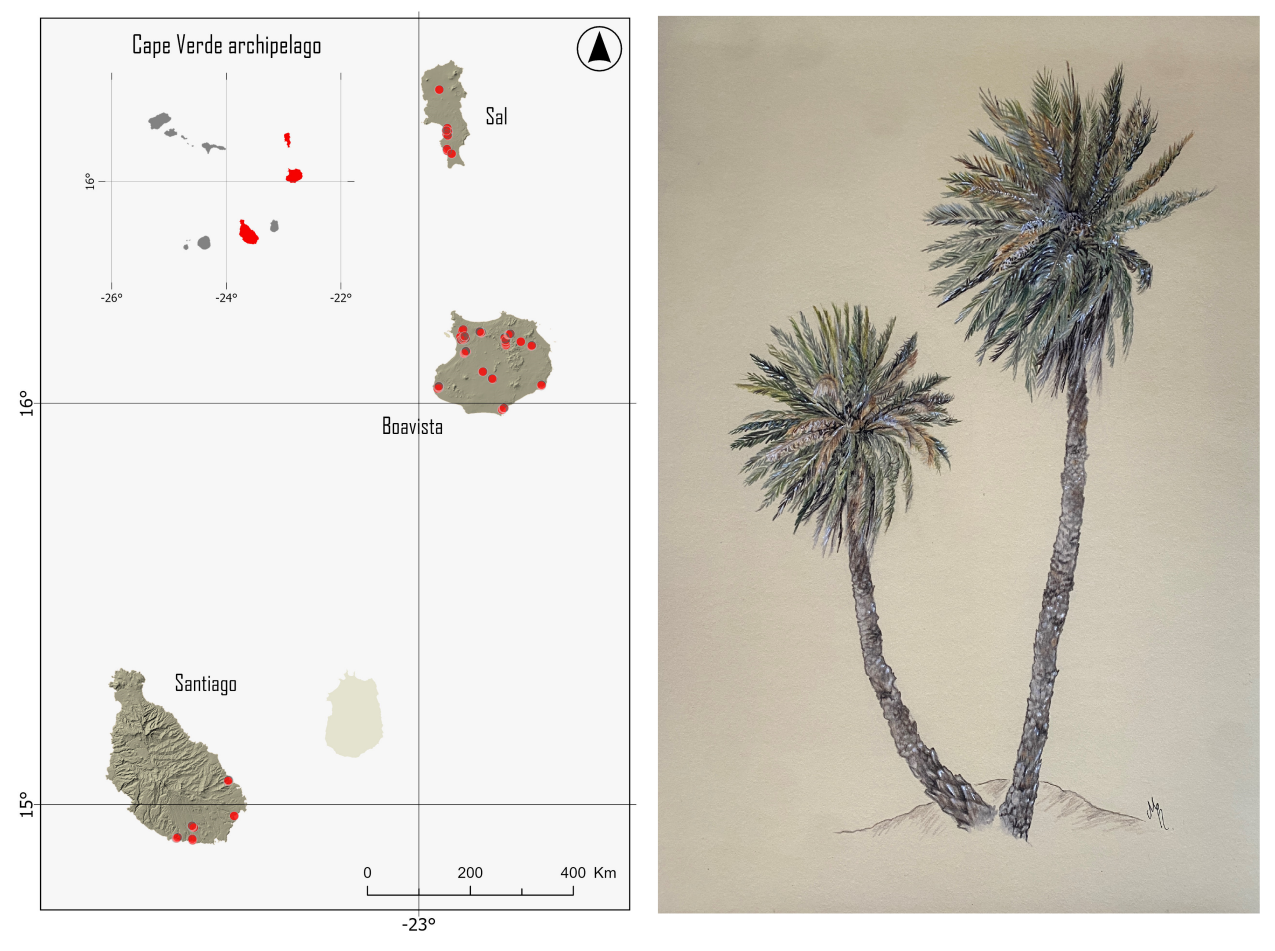
Map showing sample locations of palms collected from three islands in Cape Verde: Sal, Boavista, and Santiago (**left**). Drawing of *P. atlantica* by Guacimara Arbelo Ramírez (**right**).

The collected samples were chosen by personnel from the Direção Geral da Agricultura, Silvicultura e Pecuária e Delegação do Ministerio da Agricultura e Ambiente do Sal e da Boavista, and sampling was conducted by the research team from the University of Las Palmas de Gran Canaria (ULPGC) during their visits to Cape Verde in March (island of Sal and Santiago) and May (islands of Boavista and Santiago) 2021. Two hundred and twenty-eight specimens with morphological characteristics that corresponded to *P. atlantica* were collected from 31 locations (Figure 1 and Table S1).

Specifically, 126, 55, and 47 samples were collected from Boavista, Sal, and Santiago, respectively. For each randomly selected specimen, 3–5 leaflets were cut and stored in silica gel for the dehydration of plant tissues. All the sampled individuals were mapped using a GPS (GPSmap 76CS, Garmin, Kansas City, MO, USA) and the coordinates were projected to a universal transverse mercator coordinate system (UTM). Most of the samples were collected from the wild. However, at two locations, Viveiro Pachamama Ecopark and Viveiro Joao Galego, cultivated samples were collected. In the island of Sal, a significant portion of the specimens (N = 24) was procured from Viveiro Pachamama Ecopark, a nursery situated in the southern sector of the island. The palms in this enclosure were artificially germinated from locally collected progenitors and were first-generation palms. The owners of the nursery asserted that their palm collection included specimens of “tamareiras”, referred to locally as genuine *P. atlantica*. This sparked a strong interest in conducting genetic analyses on these specimens to evaluate their genetic composition and confirm their purity, with the goal of potentially establishing them as reliable sources for future replantation initiatives. In addition, *P. dactylifera* samples from Tunisia (N = 412) and Djibouti (N = 126) and *P. canariensis* (N = 426) were also included in the comparative analyses.

### 2.2. DNA Isolation and Microsatellite Analysis

The total genomic DNA was extracted using an Invisorb^®^ DNA Plant HTS 96 Kit (STRATEC Molecular, Berlin, Germany) following the manufacturer’s protocol. DNA aliquots were adjusted to ensure a minimum concentration of 20 ng·μL^−1^. Subsequently, a panel of 18 microsatellite simple sequence repeat (SSR) markers located in the nuclear genome and a minisatellite located in the chloroplast genome (Table S2) were amplified at ADNid (Montpellier, France). Here, the extraction and amplification of this set of molecular markers for the *Phoenix* genus are standardized and have contributed considerably to the molecular characterization of *Phoenix* [24]. These microsatellites were selected because they were previously proven to have a high-resolution power for addressing questions about hybridization between *Phoenix* specimens [6,11,20,24]. Moreover, they are transferable between *Phoenix* species [22,23]. Specifically, the amplification reactions were conducted using the Type-it Microsatellite PCR Kit (QIAGEN, Hilden, Germany), following the manufacturer’s instructions. The amplified products were analyzed using an ABI Prism 3130xl Genetic Analyzer (Thermo Fisher Scientific, Waltham, MA, USA). For the analysis, 5 μL of diluted PCR products were combined with 15 μL of a size standard in Rox dye at ADNid. In total, 4% of the samples underwent a second analysis to confirm the irregularities observed during peak reading, thus avoiding genotyping errors. The alleles were read to establish the genotyping of each sample using the GeneMapper v3.7 program (Applied Biosystems, Foster City, CA, USA).

### 2.3. Data Analysis

Analyses were performed on samples from Cape Verde exhibiting the occidental chlorotype (C242) only. To explore the differences between different *Phoenix* species, we used their genetic differences based on a matrix of genetic distances that was subsequently incorporated into a principal component analysis (PCA) using GenAlEx v6.5 [25]. The population structure was inferred using the Bayesian clustering procedure implemented in STRUCTURE v2.3.4 [26] that identifies the K (unknown) genetic clusters of origin of the sampled individuals and assigns the individuals to the inferred clusters. The algorithm uses Markov chain Monte Carlo (MCMC) to explore a parameter space considering individual memberships of the K clusters. Specifically, one million MCMC steps were executed, with a burn-in period of 10,000 iterations. The analysis considered K clusters ranging from K = 1 to K = 9. The optimum K (number of true clusters in the data) was obtained following the method described in ref. [27], implemented in STRUCTURE SELECTOR [28]. The iterations were clustered with CLUMPAK [29]. Additionally, a discriminant principal component analysis (DPCA) was performed for the *P. atlantica* and *P. dactylifera* C242 samples using *adegenet* package v2.1.10 [30] and plotted using the *ggplot2* package v3.5.1 [31] in R v4.1.2 [32].

Standard genetic diversity statistics, including the number of different alleles ($N_{a}$), number of effective alleles ($N_{e}$), number of private alleles ($N_{pa}$), observed heterozygosity ($H_{o}$), expected heterozygosity ($H_{e}$), and the average across all loci for Weir and Cockerham’s fixation index ($F_{IS}$) and $F_{ST}$, were calculated using GenAlEx v6.5. Moreover, an analysis of molecular variance (AMOVA) was performed to assess the genetic differentiation at different hierarchical levels using the *ade4* package v1.7.22 in R v4.1.2 using 1000 permutations. The AMOVA compared the genetic variation between the islands, between the populations within the islands, and the samples within the populations. A pattern of isolation by distance (IBD) within and across the three islands in Cape Verde was tested for wild samples by correlating the $F_{ST}$ values (expressed as $F_{ST}$/(1 −$F_{ST}$)) with the geographical distance (km), computed using UTM coordinates in GenAlEx v6.5. Plotting was performed using R v4.1.2. Additionally, loci deviating from Hardy–Weinberg equilibrium for each population were identified using the *pegas* package v1.3 in R v4.1.2. Furthermore, linkage disequilibrium between loci and the presence of null alleles were analyzed using the *poppr* and *PopGenReport* packages in R v4.1.2.

### 2.4. Demographic Analysis

Approximate Bayesian computation (ABC) was implemented in the DIYABC V2.0 [33] and used to infer the demographic history of *P. atlantica* with respect to *P. dactylifera* and *P. canariensis*. This approach uses coalescence tools to generate thousands of simulated data points from sets of user-defined demographic scenarios. A similarity criterion based on summary statistics from the simulated and observed data sets is applied to compare alternative scenarios and to infer the distribution parameters without explicit likelihood calculations.

To simplify the computations and provide meaningful scenarios, samples of *P. dactylifera* were grouped by location (Djibouti and Tunisian samples belong to the eastern and western pool according to [20]). Moreover, only samples assigned to the *P. atlantica* cluster based on previous analyses were included. Six scenarios were tested—see more information in Figure S3. For all the scenarios, we assumed both different effective population sizes for each population (N1, N2, N3, N4) and a different effective size of non-sampled ancestral populations (NA) following each split event (i.e., NA1 < NA2 < NA3).

Following the recommendations of ref. [34], we chose thirty-six summary statistics for the observed and simulated data sets, which were recorded as sample statistics to estimate the scenario probability and parameter confidence intervals and to assess the confidence levels for the selection of a given scenario. Specifically, the mean number of alleles, expected heterozygosity ($H_{e}$), and mean allelic size variance were used for each population. Pairwise $F_{ST}$ and the classification index were used for pairs of populations. The nuclear markers followed autosomal inheritance, while the chloroplastic minisatellite was maternally inherited, indicated as the mitochondrial inheritance in the program. One million simulations were performed for each scenario, and the most likely scenario was evaluated by comparing the posterior probabilities with the logistic regression procedure using the top 10% closest simulated points [35]. The goodness-of-fit of the four scenarios was also assessed using a principal component analysis (PCA) using the option ‘modelling checking’ in DIYABC.

## 3. Results

### 3.1. Genetic Relationship of P. atlantica with Other Phoenix Species

In this study, of the 228 analyzed specimens collected in Cape Verde, 219 harbored the occidental chlorotype (C242) and 9 had the oriental chlorotype (C254), the latter of which were excluded from the analyses (see ‘Discussion’).

**Figure 2:**
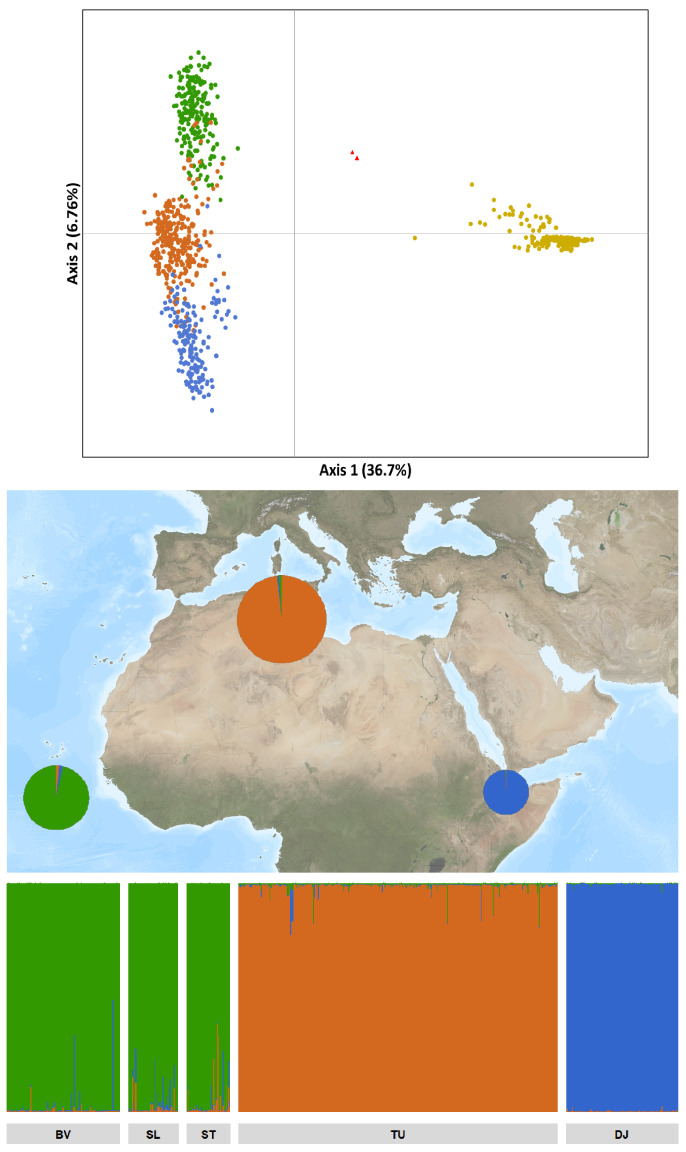
Genetic differentiation and structure of the samples studied. Above: Principal component analysis (PCA) of samples collected in Cape Verde (green), *P. canariensis* (yellow), and *P. dactylifera* C242 (orange) and C254 (blue) chlorotypes. Cape Verde samples are chlorotype C242, and *P. canariensis* samples are chlorotype C266. Two samples collected from Cape Verde, positioned between the two main clusters, are highlighted with red triangles. Middle: A map of the study region with pie charts indicating the genetic composition of samples from Cape Verde and *P. dactylifera* from Tunisia (C242) and Djibouti (C254). Pie charts are scaled proportionally to the number of samples at each location to illustrate sample size. Below: Clustering results obtained with STRUCTURE. Abbreviations: BV: Boavista; SL: Sal; ST: Santiago; TU: Tunisia; DJ: Djibouti.

The PCA results revealed a clear separate clustering of *P. dactylifera* and the samples collected in Cape Verde from *P. canariensis*, indicative of their genetic differentiation (Figure 2). Moreover, within the former group, the samples clustered in three subgroups, corresponding to samples collected in Cape Verde and the two differentiated chlorotypes of *P. dactylifera*. The samples obtained from Cape Verde were clustered closer to the samples of *P. dactylifera* with the occidental chlorotype C242. Additionally, two of the samples collected in Cape Verde positioned themselves between the *P. canariensis* and *P. dactylifera* samples from the Cape Verde cluster.

Considering the close genetic relationship between the samples collected in Cape Verde and *P. dactylifera*, we inferred the population structure between these samples using the Bayesian clustering procedure implemented in STRUCTURE. The best fit for our dataset was K = 3 (Figure S1), corresponding to three distinct clusters: samples collected in Cape Verde, date palm C242 from Tunisia, and date palm C254 from Djibouti (Figure 2).

**Figure 3:**
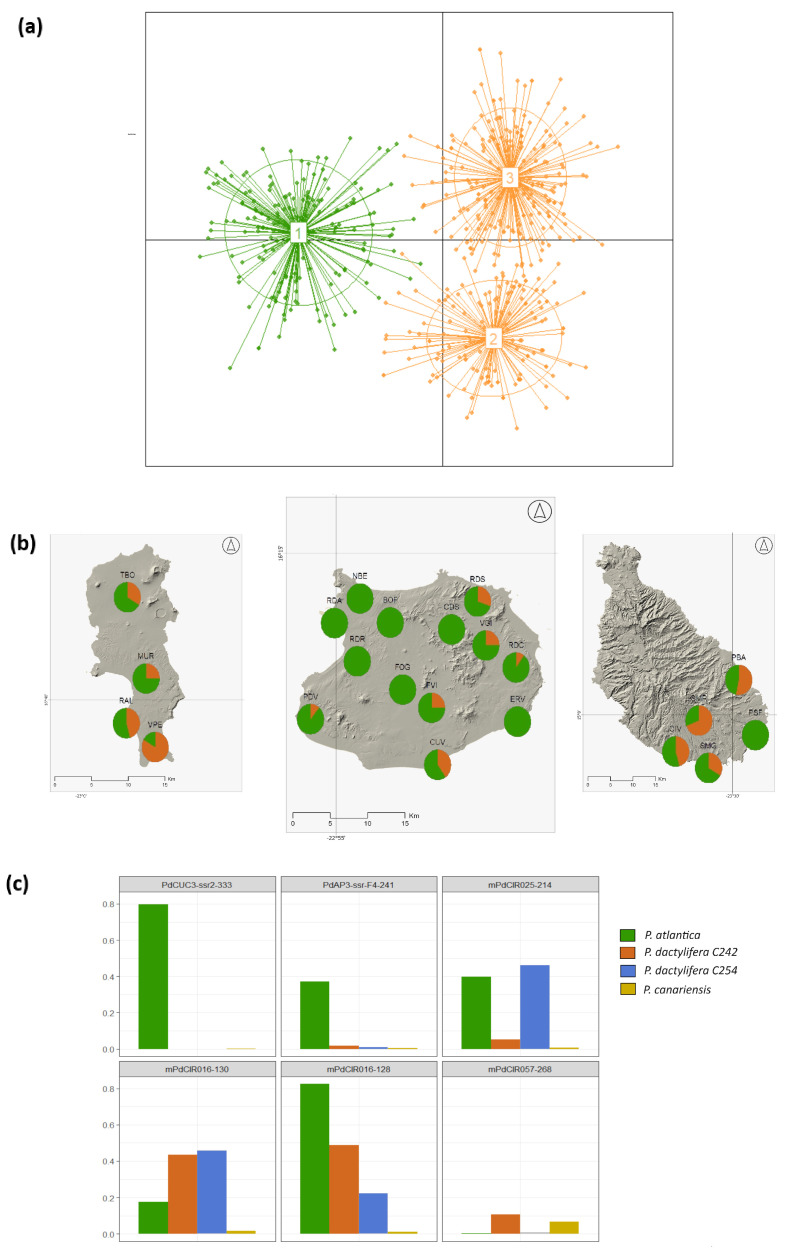
(**a**) Discriminatory principal component analysis (DPCA) of *P. atlantica* and *P. dactylifera* C242. Most samples from Cape Verde were categorized into cluster 1 (73.5%), while all samples from Djibouti fell into cluster 2. Cluster 3 is predominantly comprised samples from Tunisia, making up 86.7% of the total samples. (**b**) Proportion of individuals at each sampling site in the three islands belonging to the *P. atlantica* cluster (green) or the *P. dactylifera* cluster (orange). (**c**) Bar plot depicting the allele frequencies of the six alleles responsible for the differentiation seen in the DPCA. Specifically, the top three alleles are accountable for the variation observed along Axis 1, whereas the trio of alleles below drive the differentiation observed along Axis 2.

To identify groups within Cape Verde and *P. dactylifera* C242, we performed a DPCA. Three clusters were detected, which included one differentiated cluster composed mainly of samples collected in Cape Verde (Figure 3). Specifically, the samples collected in Cape Verde assigned to this cluster were more prevalent in Boavista (91.7%) than in Sal (54.8%) and Santiago (50%) (Figure 3). However, in Sal, when excluding the samples from the nursery (VPE in Figure 3), most of the wild samples were assigned to the *P. atlantica* cluster (79.3%). The genetic differentiation between the clusters was mainly based in the allele frequency of six alleles at five loci (Figure 3). Specifically, the separation along the first axis of the DPCA was attributed to three alleles. Notably, one allele was almost exclusively associated with *P. atlantica*, except for a single individual from *P. canariensis*. Explicitly, this individual occupied a position in the PCoA between the cluster of *P. atlantica*–*P. dactylifera* and *P. canariensis*. Additionally, the two remaining alleles were observed with a higher frequency in *P. atlantica* than in *P. dactylifera* C242 (Figure 3).

### 3.2. Demographic History

**Figure 4:**
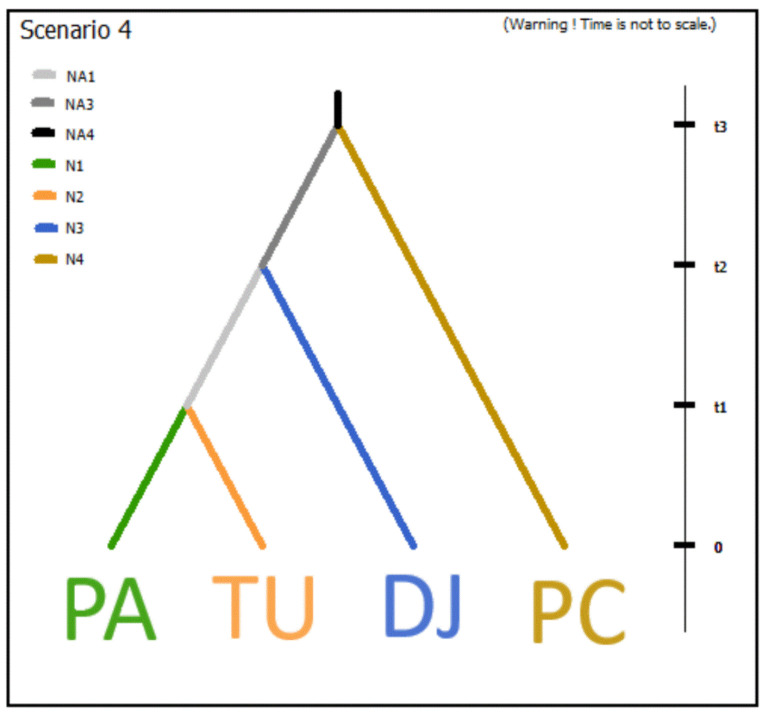
Scenario with the highest score for the population history between *P. canariensis* (PC), *P. dactylifera* (TU, DJ), and *P. atlantica* (PA). Specifically, this scenario (scenario 4) describes a sequential split of *P. canariensis*, *P. dactylifera* C254, and simultaneous divergence of *P. dactylifera* C242 and *P. dactylifera* following the ‘progression rule’ from the eastern to western populations in *P. dactylifera* and *P. atlantica*. Ti: time scale, measured in generations. Ni: effective population size of actual populations, where i ranged from 1 to 4; Nai: effective population size of non-sampled ancestral populations, where i ranged from 1 to 3 in this scenario.

The ABC results show that scenario #4, i.e., the split of *P. dactylifera* C254 from *P. canariensis* followed by a split of *P. dactylifera* C242 from *P. atlantica*, resulted in the highest estimated posterior probabilities using a logistic approach with a 10% closest point of 0.8643 (0.8643, 95% CI = 0.8170–0.9116); see Figure 4.

Considering this scenario, we inferred the posterior distribution of the parameters for this model (Table S3). Specifically, the split of *P. atlantica* and *P. dactylifera* from Tunisia was approximately 840 generations ago (95% CI = 169–3600). Estimates of the reproductive age of date palm have been previously determined to be 4–9 years [36]. Assuming such estimates for generation time, the divergence time between *P. atlantica* and *P. dactylifera* could have occurred 3360–7560 years before the present (YBP).

Regarding the estimated effective population sizes for the sampled populations, the median values ranged from 19,240 (*P. dactylifera* C254 from Djibouti) to 1810 (*P. atlantica*). Moreover, both *P. atlantica* and *P. dactylifera* C242 from Tunisia have decreased their effective size with respect to their ancestral population (NA1 = 32,000).

### 3.3. Characterization of P. atlantica in Cape Verde

**Figure 5:**
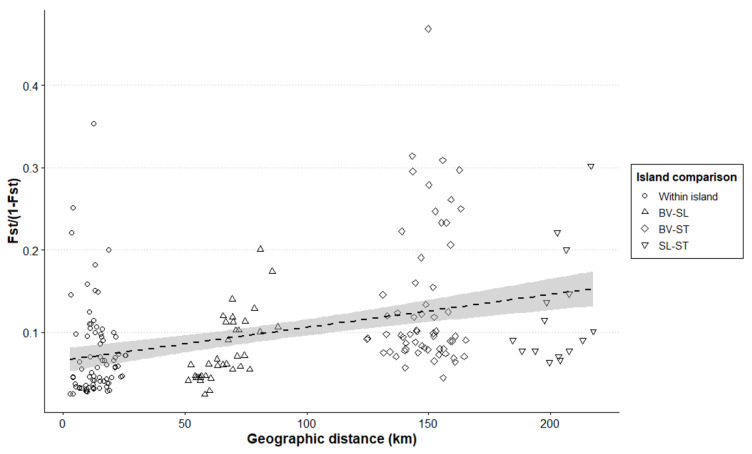
Isolation by distance (IBD) of *P. atlantica* within and between the islands of Cape Verde.

The genetic diversity of the samples assigned as *P. atlantica* is summarized in Table S4. Overall, the genetic diversity was heterogeneous among the populations, with values of expected heterozygosity ranging from 0.158 to 0.446. At the island level, Santiago has the highest number of private alleles, with 10 private alleles, 9 of which had frequency higher than than 12%. Boavista had 7 private alleles, followed by Sal with 4 private alleles. Furthermore, *P. atlantica* in Cape Verde had low differentiation among the islands, with some genetic differentiation between Boavista and Sal/Santiago under K = 2 (Figure S4). Accordingly, the AMOVA results (Table S5) show that the genetic variation between the islands accounts for only 2.92% of the total variation (Phi = 0.03). The variation between the populations within the islands is slightly higher, contributing to 4.94% of the total variation (Phi = 0.05), while the genetic variation of the samples within the populations is negative (−2.08%, Phi = −0.02). Despite the low genetic variation explained by the differences between the islands, we observed an IBD pattern in *P. atlantica* (Figure 5).

Additionally, we compared the genetic diversity and differentiation of *P. atlantica* to *P. canariensis* and *P. dactylifera* species (Tables S6 and S7). *P. dactylifera* had the highest genetic diversity, followed by *P. atlantica* and *P. canariensis*. The genetic differentiation between the species was lowest between *P. atlantica* and *P. dactylifera* C242 ($F_{ST}$ = 0.16) and highest between *P. atlantica* and *P. canariensis* ($F_{ST}$ = 0.59), followed by *P. dactylifera* C254 and *P. canariensis* ($F_{ST}$ = 0.56). *P. atlantica* exhibited a private allele at one locus. Additionally, *P. dactylifera* C242 displayed a private allele at the same locus as the private allele of *P. atlantica*. *P. canariensis* showed fixation of private alleles exceeding 97% in five nuclear alleles and the chloroplast marker.

## 4. Discussion

This study characterizes *P. atlantica* populations in the Cape Verde archipelago and provides insights into their intricate relationships with their relative *P. dactylifera*.

### 4.1. Genetic Characterization of P. atlantica

The genetic analysis of *P. atlantica* in this study uses a larger sample size compared to previous studies [11,13,14]. This increased sample size significantly enhances our ability to accurately characterize *P. atlantica*, whose differentiation has been recently disputed. The larger sample size is critical due to the morphological similarity between *P. atlantica* and *P. dactylifera*, which could potentially have lead to sample misidentification in previous molecular analyses.

Our analyses revealed variations in the allele frequencies across six alleles and five loci, highlighting a unique genetic profile for *P. atlantica* compared to *P. dactylifera*. Despite using the same genetic markers, we did not identify as many private alleles for *P. atlantica* compared to those reported in ref. [11]. However, contrary to ref. [14], we did identify one fixed private allele in *P. atlantica*. This unique allele was present in approximately 80% of the *P. atlantica* individuals and was absent in the other groups, except for a single *P. canariensis* individual, where it appeared in the heterozygous form. This particular *P. canariensis* specimen exhibited both the nuclear chlorotype 266 and a duplication in the Pd15 loci; a genetic trait associated with *P. canariensis* [8]. This raises the possibility that this sample might have been misidentified as *P. canariensis*, when it could, in fact, be a hybrid with *P. atlantica*. Additionally, our results indicate a different private allele in the same loci as *P. atlantica* in *P. dactylifera* (Table S6), suggesting possible genetic selection between *P. atlantica* and *P. dactylifera*, despite their close genetic relationship and shared ancestry.

*P. atlantica* exhibited low genetic differentiation among the islands, with a higher level of coancestry observed in one of the clusters on Boavista. However, caution is warranted in interpreting these results due to the potential influence of the larger sample size from Boavista, which could exaggerate the observed genetic differentiation between the islands in *P. atlantica*, as well as the bias towards K = 2 in the genetic structure analysis [37]. Additionally, the AMOVA results indicated a small variance among the islands or the populations within the islands. The slightly greater variation between the populations within the islands compared to the islands suggests differentiation at a finer spatial scale. Moreover, the lack of significant substructuring among the samples within the same population is likely due to high gene flow. Furthermore, the observed limited genetic structuring in *P. atlantica* suggests a scenario of recent divergence or ongoing population expansion, potentially limiting the emergence of distinct genetic patterns. This hypothesis is supported by the recent divergence of *P. atlantica* from *P. dactylifera* observed in this study.

Regarding genetic diversity, *P. atlantica* showed a lower diversity compared to *P. dactylifera*. Similarly, *P. canariensis* also exhibited a low genetic diversity, which may be a consequence of the isolated and fragmented populations typically found on islands [38]. Consistent with this observation, *P. atlantica* showed a reduced effective population size compared to its ancestral population. Additionally, both *P. atlantica* and *P. canariensis* demonstrated a notable degree of IBD. *P. canariensis* has previously been reported to disperse via the stepping-stone method within the Canarian archipelago [21]. This phenomenon aligns with other species on oceanic islands, which have also been documented to experience genetic differentiation with distance [39,40].

Additionally, our results revealed differences in the distribution of *P. atlantica* across various islands within the Cape Verde archipelago. Boavista and Sal emerged as hosts to the largest populations of wild *P. atlantica*, while the prevalence of *P. dactylifera* individuals in Sal’s nursery suggested deliberate or unintentional human introduction of date palms in this specific enclosure. In contrast, Santiago exhibited the highest number of wild individuals classified as *P. dactylifera*. The earliest populated island in the early 15th century, Santiago served as the archipelago’s inaugural political–administrative division and housed the pioneering Portuguese settlement of Cidade Velha, marking its significance as Sub-Saharan Africa’s first Portuguese outpost and a pivotal stopover in transatlantic voyages [41]. This early history of colonization likely rendered Santiago more susceptible to date palm introductions, thereby contributing to the observed genetic composition patterns of the palms between islands. Additionally, high tourism rates could have facilitated date palm introductions in the archipelago [19]. Nevertheless, the presence of tourism alone does not fully explain the observed variations between the islands, given that all the islands considered in our research experience similar levels of tourism. Additionally, our study only covered three islands; therefore, the Cape Verde palms on the remaining islands remain uncharacterized.

### 4.2. P. atlantica Is Closely Related to P. dactylifera from North Africa

Our results indicate a close genetic relationship between *P. atlantica* and *P. dactylifera*, consistent with previous studies [2,12]. Firstly, most of the samples collected in Cape Verde had chlorotype C242, shared with *P. dactylifera* from Tunisia, in line with earlier literature [14,20,42]. Only a few samples from Cape Verde had the oriental chlorotype C254, and their clustering with *P. dactylifera* C254 in the PCoA and DPCA analyses (not shown) suggest that these specimens likely represented recently introduced *P. dactylifera* rather than native individuals.

The close genetic relationship between *P. atlantica* and *P. dactylifera* in North Africa was also observed in our simulation analysis, which supports a recent divergence between these two groups. The observed genetic divergence aligns with the widely accepted hypothesis tracing the evolutionary lineage of *P. atlantica* back to *P. dactylifera* [10,14]. Moreover, both the Tunisian date palms and *P. atlantica* showed a reduction in their effective population size compared to their shared ancestral population. This decline is likely due to a founder effect, as evidenced by the significant effective population size seen in the Djibouti date palm population, which is closely related to the wild and ancestral date palms from Oman [13,14]. Similarly, *P. canariensis*, like *P. atlantica*, showed a reduction in its effective population size relative to its ancestral population. This aligns with the founder effect and stepping-stone dispersal previously described for *P. canariensis* in the Canary Islands [21].

Regarding the divergence timeline of *P. atlantica* from date palms (3360–7560 YBP), our simulations indicate that this occurred prior to human presence on the island and Portuguese colonization in the 15th century [41]. This suggest the potential initial natural dispersion of the palms from mainland Africa to Cape Verde. Similarly, *P. canariensis* reached the Canary Islands before human colonization [43], supporting the hypothesis that either *P. atlantica* or *P. dactylifera* dispersed naturally to Cape Verde from mainland Africa over a short waterway after or before their divergence into separate groups, respectively. However, converting generations into precise years remains challenging because it depends on a range of generations and reproductive ages. Therefore, determining whether *P. atlantica* or *P. dactylifera* reached Cape Verde naturally requires further, more precise analysis. While we cannot definitively determine whether the palms arrived naturally or were introduced by humans in the archipelago, our results show that *P. atlantica* diverged recently from *P. dactylifera*, supporting previous research demonstrating that *P. atlantica* might be a feral form of the date palm [12,13,14].

Additionally, our model indicates that the Djibouti population diverged earlier than the date palms from Tunisia. This finding is consistent with previous research suggesting that Middle Eastern populations retain 80% of the genetic variation present in wild date palms from Oman [14]. Furthermore, archaeological evidence suggests a delayed appearance of date palms in North Africa [44,45] compared with the Middle East [46,47], implying the expansion of the Middle Eastern date palm’s natural range to the African continent [13,48]. Therefore, our results support a scenario that is consistent with previous research findings.

Overall, our results provide insight into a recent divergence between *P. atlantica* and *P. dactylifera*, enhancing our comprehension of the complex historical paths of palm trees in Africa. However, the exact date of divergence in our models should be viewed with caution. For a more accurate estimation of the divergence timeline, further analysis should include date palm populations that are geographically closer to Cape Verde, consider the ancestral wild population from Oman [14], and include more markers in the chloroplast.

### 4.3. Implications for P. atlantica Conservation

Due to its genetic differentiation, it is essential to preserve the unique genetic pool of *P. atlantica*. This is particularly important because the genetic composition of this palm tree varies between islands and exhibits a pattern of IBD. Thus, we emphasize the importance of implementing a seed and/or germplasm bank using wild samples from Boavista and Sal as an ex situ conservation method. For this purpose, we discourage the use of samples from the nursery in Sal as a potential source for future replantation initiatives. Furthermore, in line with our results regarding the patterns of genetic differentiation between *P. atlantica* populations, we recommend prioritizing local sourcing within nearby individuals to maintain natural dispersal mechanisms and connectivity between populations. Specifically, the IBD pattern observed in *P. atlantica* enhances conservation efforts by identifying critical protection areas, thus safeguarding the genetic diversity that is crucial for species’ long-term survival in their natural habitats, as previously supported by landscape genetic research [49].

Moreover, we must also consider the implications of climate change on future landscapes in Cape Verde. Presently, *P. atlantica* predominantly inhabits low elevations, where the average annual temperature ranges from 20.9 to 25 °C, with maximum annual precipitation of 271 mm [17]. According to climate models, the habitat suitability for *P. atlantica* is projected to decrease in the northern parts of Sal and Santiago, remain relatively stable in the south of these islands, and increase in Boavista [17]. These future scenarios should guide decisions on replantation, thereby enhancing the efficacy of conservation endeavors. Moreover, integrating these insights into strategic planning for establishing protected areas, especially in regions like Boavista where such zones are currently lacking, is recommended to protect habitats suitable for *P. atlantica*.

The identified fixed private allele in *P. atlantica* hints at its potential role as a genetic marker for differentiating this species from *P. dactylifera* in a wider investigation across Cape Verde. However, replication studies are necessary to confirm the uniqueness of the identified allele in *P. atlantica* and to determine its prevalence across different populations, including those on other islands. Additionally, these studies should aim to identify additional alleles to establish a more comprehensive set of genetic markers, such as the characterization of genetic markers performed for *P. canariensis* [8].

## 5. Conclusions

Our study reveals markers responsible for the genetic differentiation between *Phoenix atlantica* and *Phoenix dactylifera*, despite their recent evolutionary divergence and morphological similarity. Additionally, we conducted a comprehensive genetic characterization of palm tree populations across three islands in the Cape Verde archipelago; an effort not previously documented in the scientific literature to our knowledge. These findings underscore the existence of a unique genetic pool and provide essential insights into the genetic composition palm tree populations in Cape Verde, which are crucial for informing conservation strategies. Furthermore, the identified fixed allele holds potential as a genetic marker for distinguishing *P. atlantica* from other *Phoenix* species in broader studies across Cape Verde, offering prospects for discovering additional distinguishing markers. However, further research including comprehensive morphological, morphometric, and genetic studies is essential to clarify the taxonomic status of *P. atlantica*.

## Data Availability

The original contributions presented in the study are included in the Supplementary Materials, further inquiries can be directed to the corresponding authors.

## Supplementary Materials

The following supporting information can be downloaded at: https://www.mdpi.com/article/10.3390/plants13162209/s1, Table S1: Number of samples collected from Cape Verde with chlorotype C242 in three islands: Boavista, Sal, and Santiago (refer to Figure 1). Samples are grouped by their respective location. Table S2: Description of the polymorphic nuclear microsatellites (nSSR) used in this study. F: Forward primer. R: Reverse primer. Motif: sequence that is repeated in tandem a variable number of times. Range: Range of sizes in number of base pairs (bp) that show the amplified fragments from each nSSR. Table S3: Posterior distributions of parameters in Scenario #3. Scenarios in this analysis are shown in Figure S3. Table S4: Location and summary of genetic diversity estimates obtained with 18 microsatellites for *P. atlantica* (N = 151) in each population and island for studied populations: number of samples per population (N), number of different alleles (Na), number of effective alleles (Ne), number of private alleles (Npa), observed heterozygosity (Ho), unbiased expected heterozygosity (He) and their respective standard error (SE). Table S5. Analysis of Molecular Variance (AMOVA) results. The findings indicate that there is minimal variance between different populations or islands, with the majority of the observed variance occurring within individuals. The estimated variance attributed to differences among individuals within populations is negative, likely due to the extremely small values involved. Table S6: Genetic diversity estimates obtained with 18 microsatellites for *P. atlantica*, *P. dactylifera* C242, *P. dactylifera* C254 and *P. canariensis* in each population and island for studied populations: number of different alleles (Na), number of effective alleles (Ne), number of private nuclear alleles with a frequency higher than 70% (Npa), observed heterozygosity (Ho), unbiased expected heterozygosity (He) and their respective standard error (SE). The nuclear private alleles listed in the Npa column, and their frequencies are detailed in the last column. Table S7: Pairwise genetic differentiation (FST) between Phoenix species using AMOVA with 999 permutations. All FST values were found to be significant at *p* < 0.001. Figure S1. DeltaK and Mean L(K) Probability from STRUCTURE Analysis and Clustering Analysis Results. Above: The results indicate that K = 3 is the best fit for *P. atlantica* and *P. dactylifera* C242 and C254. Below: Results of structure analysis. Three distinct groups are supported by the analysis including samples from Cape Verde (BV, SL, ST) in green, samples from *P. dactylifera* C242 (TU C242, DJ C242) in orange and *P. dactylifera* C254 (DJ C254, TU C254) in blue. Samples C242 from Djibouti show some admixture with *P. dactylifera* C254 group and Samples C254 from Tunisia show admix-ture with all groups. Figure S2. Loading plot for first (above) and second (below) axis in the Discriminant Principal Component Analysis (DPCA). Figure S3. Six scenarios of population demography of *P. atlantica*, *P. dactylifera* and *P. canariensis* examined by ABC analysis as implemented in DIYABC v2.0 (Cornuet et al., 2014): a common split for all populations (scenario #1); ancestral divergence of two genetic pools (*P. atlantica* and *P. dactylifera* vs. *P. canariensis*) and subsequent split into different populations of *P. dactylifera* and *P. atlantica* (scenario #2); sequential split of *P. canariensis*, *P. atlantica* and simultaneous divergence of *P. dactylifera* (C242 and C254) (scenario #3); sequential split of *P. canariensis*, *P. dactylifera* C254 and simultaneous divergence of *P. dactylifera* C242 and *P. atlantica*, following the ‘progression rule’ from eastern to western populations in *P. dactylifera* and *P. atlantica* (scenario #4); ancestral divergence of two genetic pools (*P. atlanticas* *P. dactylifera* and *P. canariensis*) and subsequent split into different populations of *P. dactylifera* and *P. canariensis* (scenario #5); and, lastly sequential split of *P. atlantica*, *P. canariensis* and simultaneous divergence of *P. dactylifera* (C242 and C254). The samples were considered in the scenarios as each sampled population: Pop 1: *P. atlantica* from Cape Verde; Pop 2; *P. dactylifera* C254 from Djibouti, Pop 3: *P. canariensis* and Pop 4: *P. dactylifera* C242 from Tunisia. Ti time scale is measured in generations, where i ranged from 1 to 4; Ni effective population size of actual populations, where i ranged from 1 to 5; Nai effective population size of non-sampled ancestral populations, where i ranged from 1 to 4. Figure S4. DeltaK and Mean L(K) Probability from STRUCTURE Analysis and Clus-tering Analysis Results. Above: The results indicate that K = 2 is the best fit for *P. atlantica*, with K = 4 as the next best fit. Below: The results of the structure analysis. At K = 2, there is genetic structure between BV, SL, and ST. However, at K = 4, no clear pattern emerges, and the genetic composition of individuals appears mixed.
