# Supplementary material for: A Comparative Genetic Analysis of Phoenix atlantica in Cape Verde
Source: Plants (Basel). 2024 Aug 9;13(16):2209. doi: 10.3390/plants13162209 (PMC11360615; doi:10.3390/plants13162209)
Supplement: Supplementary file 1 [file plants-13-02209-s001.zip › plants-3050936-supplementary.pdf]

A Comparative Genetic Analysis of Phoenix atlantica in Cape Verde

Sonia Sarmiento Cabello[1,*] , Priscila Rodriguez-Rodriguez[1], Guacimara Arbelo Ramirez[1], Agustin Naranjo-Cigala[1], Leticia Curbelo[1], Maria de Monte da Graca Gomes[2], Juliana Brito[2], Frederique Aberlenc[3], Salwa Zehdi-Azouzi[4] and Pedro A. Sosa[1,*]

1 Instituto Universitario de Estudios Ambientales y Recursos Naturales (IUNAT), Universidad de Las Palmas de Gran Canaria, Campus Universitario de Tafira, 35017 Las Palmas de Gran Canaria, Spain

2 Direção Geral Da Agricultura Silvicultura e Pecuaria e Delegação do Ministerio da Agricultura e Ambiente do Sal e da Boavista, Praia, Cabo Verde.

3 Plant Diversity, Adaptation and Development, Université de Montpellier, Institut de Recherche pour Développement, Centre de Coopération Internationale en Recherche Agronomique pour le Développement, 911 Av. Agropolis, BP 64501, 34394 Montpellier CEDEX 5, France

4 Laboratoire de Génétique Moléculaire, Faculté des Sciences de Tunis, Immunologie et Biotechnologie (LR99ES12), Université de Tunis El Manar, Campus Universitaire Farhat Hached, Tunis 1068, Tunisia

* Correspondence: sonia.sarmiento@ulpgc.es (S.S.C.); pedro.sosa@ulpgc.es (P.A.S.)

E-mail sonia.sarmiento@ulpgc.es and Pedro.sosa@ulpgc.es

Tel.: +34 928 454550; Fax: + 34 928 452922

Table S1: Number of samples collected from Cape Verde with chlorotype C242 in three islands: Boavista, Sal, and Santiago (refer to Figure 1). Samples are grouped by their respective location.

Location	Location code	Number of samples
Boavista	BV	120
Ervatão	ERV	11
Bofareira	BOF	8
Campo da Serra	CDS	17
Curral Velho	CUV	10
Fogão	FOG	8
Fonte Vicent	FVI	4
Natural Park da Boa Esperança	NBE	5
Ribeira Calhau	RDC	10
Ribeira d’ Agua	RDA	14
Ribeira do Rabil	RDR	6
Ribeira Scriber	RDS	10
Varandinha	PDV	9
Viveiro Joao Galego	VGI	8
Sal	SL	53
Murdeira (Biodiversity Project).	MUR	12
Ribeira do Algodoeiro	RAL	11
Terra Boa	TBO	6
Viveiro Pachamama EcoPark	VPE	24
Santiago	ST	46
Cidade Velha	CIV	11

Praia Baixo	PBA	15
Praia San Francisco	PSF	4
São Martinho Grande	SMG	3
São Martinho Pequeno	SMP	13

Table S2: Description of the polymorphic nuclear microsatellites (nSSR) used in this study. F: Forward primer. R: Reverse primer. Motif: sequence that is repeated in tandem a variable number of times. Range: Range of sizes in number of base pairs (bp) that show the amplified fragments from each nSSR.

Name	Code	5'->3' Primers	Motif	Range (pb)	Reference
mPdCIR10	Pd10	F: ACCCCGGACGTGAGGTG R: CGTCGATCTCCTCCTTT-GTCTC	$(GA)_{22}$	118–161	Billote et al., (2004)
mPdCIR15	Pd15	F: AGCTGGCTCCTCCCTTCTTA R: GCTCGGTTGGACTT-GTTCT	$(GA)_{15}$	120–156	Billote et al., (2004)
mPdCIR16	Pd16	F: AGCGG-GAAATGAAAAGGTAT R: ATGAAAAC-GTGCCAAATGTC	$(GA)_{14}$	130–138	Billote et al., (2004)
mPdCIR25	Pd25	F: GCACGA-GAAGGCTTATAGT	$(GA)_{22}$	199–231	Billote et al., (2004)

Name	Code	5'->3' Primers	Motif	Range (pb)	Reference
		R: CCCCTCATTAGGATTC-TAC			
mPdCIR32	Pd32	F: CAAATCTTTGCCGTGAG<br>R: GGTGTGGAG-TAATCATGTAGTAG	$(GA)_{19}$	284–305	Billote et al., (2004)
mPdCIR35	Pd35	F: ACAAACGGCGATGG-GATTAC<br>R: CCGCAGCTCACCTCTTC-TAT	$(GA)_{15}$	175–221	Billote et al., (2004)
mPdCIR57	Pd57	F: AAGCAGCAGCCCTTCCG-TAG<br>R: GTTCTCAC-TCGCCCAAAAATAC	$(GA)_{20}$	251–278	Billote et al., (2004)
mPdCIR63	Pd63	F: CTTTTATGTGGTCTGA-GAGA<br>R: TCTCTGATCTT-GGGTTCTGT	$(GA)_{17}$	121–156	Billote et al., (2004)
mPdCIR78	Pd78	F: TGGATTTCCATTGTGAG<br>R: CCCGAAGAGACGCTATT	$(GA)_{13}$	117–152	Billote et al., (2004)
mPdCIR85	Pd85	F: GAGA-GAGGGTGGTGTTATT<br>R: TTCATCCAGAAC-CACAGTA	$(GA)_{29}$	152–183	Billote et al., (2004)

Name	Code	5'->3' Primers	Motif	Range (pb)	Reference
mPdIRD13	P13	F: GCGGAGACAGGA-GATGGTAA<br>R: CTT-GACTGCTTCTGCTGCTG	$(CAC)_6$	198–227	Aberlenc-Bertossi et al., (2014)
mPdIRD31	P31	F: GCAGGTGGACTG-CAAAATCT<br>R: CTATTGGGGTGCTGATCCAT	$(CCA)_7$	343–372	Aberlenc-Bertossi et al., (2014)
mPdIRD33	P33	F: GGAGCATACAG-TGGGTTTGC<br>R: CAGCCTGGGAATGAG-GATAG	$(CAG)_7$	189–213	Aberlenc-Bertossi et al., (2014)
mPdIRD40	P40	F: GAGA-GATGCGTCAGGGAATC<br>R: CCAGAATCTTCCAA-GCAAGC	$(CCAGTG)_4$	175–211	Aberlenc-Bertossi et al., (2014)
PdAG1ssr	AG1	F: TCTGAT-TTCGTTTACTTCTTAGGA<br>R: TTCATATTCAGTT-GTCGGGTGTA	(GA)	260	Ludeña et al., (2011)
PdAP3ssrF4	AP3	F: GAGAAATAGA-GAGCTGTGCAAG	$(GA)_{25}$	331	Zehdi-Azouzi et al., (2015)

Name	Code	5'->3' Primers	Motif	Range (pb)	Reference
		R: CTGCAGTACTCGGA-GAACTTG			
PdCUC3ssr1	CUC3-1	F: CGTGGACTCATGACTCG-CATGTCC<br>R: GGTCCTT-GCCGGTGGCCTTC	$(GT)_{14}$	330	Zehdi-Azouzi et al., (2015)
PdCUC3ssr2	CUC3-2	F: ACATTGCTCTTTT-GCCATGGGCT<br>R: CGAG-CAGGTGGGGTTCGGGT	$(GA)_{22}$	350	Zehdi-Azouzi et al., (2015)

Table S3: Posterior distributions of parameters in Scenario #3. Scenarios in this analysis are shown in figure S3.

Parameter	Median value	q(0.05)	q(0.95)
N1 (*P. atlantica*)	1,810	515	7,630
N4 (*P. dactylifera* C242)	6,950	1,920	27,700
N2 (*P. dactylifera* C254)	19,240	7,670	39,400
N3 (*P. canariensis*)	3,910	1,970	6,940
t1	840	169	3,600
t2	2,090	636	7,240
t3	35,500	13,700	56,400
Na1	32,000	5,730	55,300
Na3	7,330	803	36,900

Parameter	Median value	q(0.05)	q(0.95)
Na4	34,700	5,470	79,100

Table S4: Location and summary of genetic diversity estimates obtained with 18 microsatellites for *P. atlantica* (N=151) in each population and island for studied populations: number of samples per population (N), number of different alleles (Na), number of effective alleles (Ne), number of private alleles (Npa), observed heterozygosity (Ho), unbiased expected heterozygosity (He) and their respective standard error (SE).

Island	Population	N	Na	Ne	Npa	Ho	He
Boavista	Arvatão	11	2.47	1.706	0	0.325 (0.06)	0.333 (0.054)
Boavista	Bofareira	7	2.58	1.947	0	0.376 (0.073)	0.354 (0.064)
Boavista	Campo da Serra	17	3.16	2.178	0	0.443 (0.069)	0.415 (0.064)
Boavista	Corral Velho	6	3.00	2.28	0	0.439 (0.072)	0.442 (0.066)
Boavista	Fogão	8	2.58	1.968	0	0.388 (0.072)	0.371 (0.062)
Boavista	Fonte Vicent	3	1.947	1.71	0	0.439 (0.099)	0.295 (0.065)
Boavista	Natural Park da Boa Esperança	5	2.474	2.092	0	0.421 (0.084)	0.376 (0.067)
Boavista	Ribeira Calhau	9	2.68	2.02	1	0.462 (0.089)	0.389 (0.064)
Boavista	Ribeira d'Agua	15	2.95	1.94	2	0.411 (0.07)	0.39 (0.057)

Island	Population	N	Na	Ne	Npa	Ho	He
Boavista	Ribeira do Rabil	6	2.68	1.97	1	0.482 (0.079)	0.388 (0.061)
Boavista	Ribeira Scriber	7	2.42	1.93	1	0.414 (0.074)	0.363 (0.063)
Boavista	Varandinha	9	2.90	2.085	1	0.4 (0.071)	0.397 (0.064)
Boavista	Viveiro Joao Galego	6	2.842	2.198	1	0.465 (0.087)	0.394 (0.069)
Sal	Murdeira (MH Biodiversity Project)	9	2.895	1.833	1	0.374 (0.074)	0.356 (0.056)
Sal	Ribeira Algo-doeiro (wild)	6	2.474	1.873	0	0.404 (0.076)	0.381 (0.058)
Sal	Terra Boa (wild)	4	2.105	1.799	0	0.487 (0.096)	0.339 (0.061)
Sal	Viveiro Pacha-mama EcoPark	5	3	2.252	3	0.474 (0.074)	0.446 (0.059)
Santiago	Cidade Velha	6	2.895	2.082	3	0.404 (0.062)	0.436 (0.057)
Santiago	Praia Baixo	7	2.632	1.819	2	0.381 (0.071)	0.36 (0.055)
Santiago	Praia San Fran-cisco	4	2.474	1.953	5	0.395 (0.077)	0.385 (0.061)
Santiago	São Martinho Grande	2	1.316	1.316	0	0.316 (0.11)	0.158 (0.055)
Santiago	São Martinho Pequeno	4	2.316	1.982	0	0.434 (0.076)	0.393 (0.059)

Table S5. Analysis of Molecular Variance (AMOVA) results. The findings indicate that there is minimal variance between different populations or islands, with the majority of the observed variance occurring within individuals. The estimated variance attributed to

differences among individuals within populations is negative, likely due to the extremely small values involved.

Group	Df	Sum Sq	Mean Sq	Variance percentage (%)	Phi
**Between islands**	2	60.34	30.17	2.92	0.03
**Between populations within islands**	19	249.45	13.12	4.94	0.05
**Samples within population**	129	968.81	7.51	-2.08	-0.02

Table S6: Genetic diversity estimates obtained with 18 microsatellites for *P. atlantica*, *P. dactylifera* C242, *P. dactylifera* C254 and *P. canariensis* in each population and island for studied populations: number of different alleles (Na), number of effective alleles (Ne), number of private nuclear alleles with a frequency higher than 70% (Npa), observed heterozygosity (Ho), unbiased expected heterozygosity (He) and their respective standard error (SE). The nuclear private alleles listed in the Npa column, and their frequencies are detailed in the last column.

Population	Na	Ne	Npa	Ho (±SD)	He (±SD)	Private alleles (Locus | Allele | Frequency)		
P. atlantica	4.3	2.1	1	0.393 (0.065)	0.403 (0.064)	PdCUC3-ssr2	333	0.8
P. dactylifera C242	7.2	3.1	1	0.504 (0.068)	0.531 (0.069)	PdCUC3-ssr2	207	0.99
P. dactylifera C254	7.6	3.3	0	0.509 (0.073)	0.519 (0.075)	NA	NA	NA
P. canariensis	6.3	2.6	5	0.210 (0.048)	0.352 (0.077)	mPdCIR015A	119	0.98
						mPdCIR015B	151	0.99
						mPdCIR016	111	0.98
						mPdCIR085	149	0.78

Population	Na	Ne	Npa	Ho (±SD)	He (±SD)	Private alleles (Locus | Allele | Frequency)		
						PdAP3-ssr-F4	229	0.99

Table S7: Pairwise genetic differentiation (F_{ST}) between *Phoenix* species using AMOVA with 999 permutations. All F_{ST} values were found to be significant at p <0.001.

P. atlantica	***P. dactylifera* C242**	***P. dactylifera* C254**	***P. canariensis***	
0	-	-	-	***P. atlantica***
0.16	0	-	-	***P. dactylifera C242***
0.24	0.26	0.000	-	***P. dactylifera C254***
0.59	0.55	0.56	0.000	***P. canariensis***

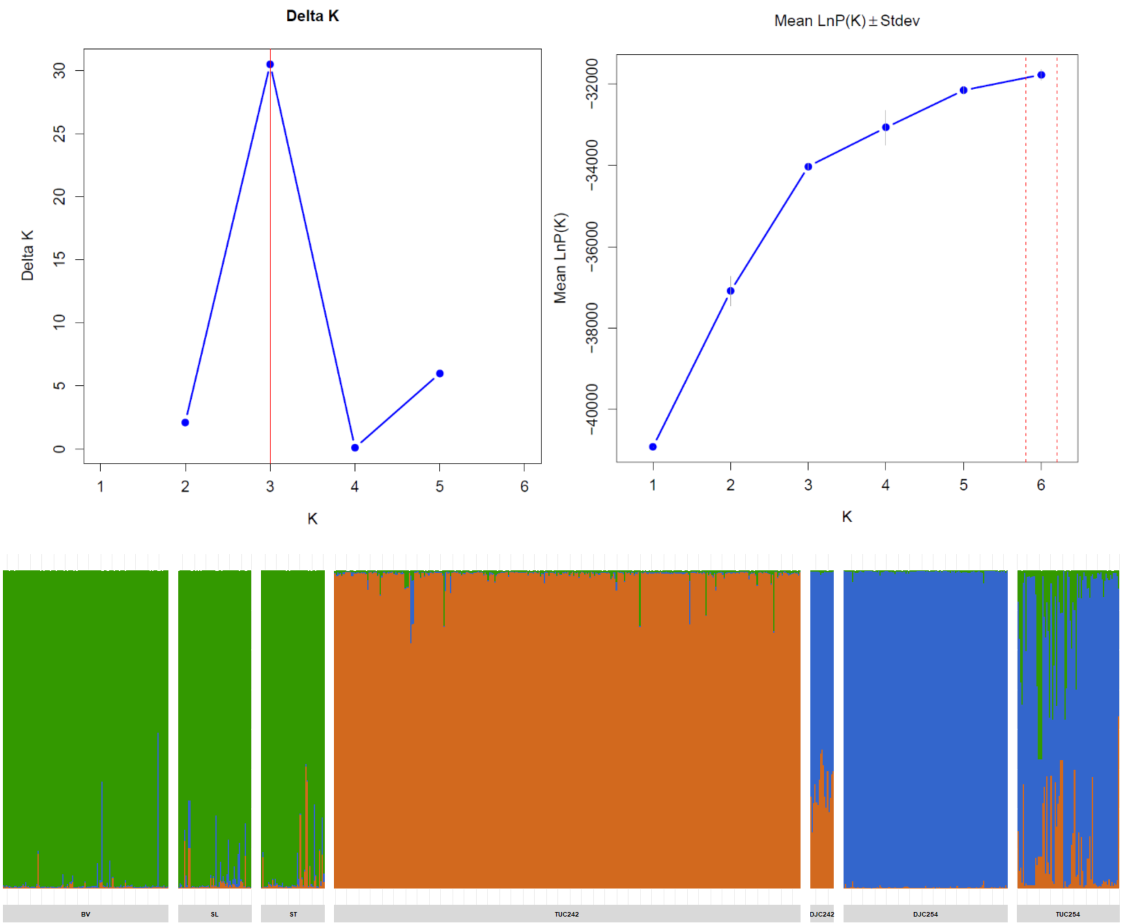

Figure S1. DeltaK and Mean L(K) Probability from STRUCTURE Analysis and Clustering Analysis Results. **Above:** The results indicate that K=3 is the best fit for *P. atlantica* and *P. dactylifera* C242 and C254. **Below**: Results of structure analysis. Three distinct groups are supported by the analysis including samples from Cape Verde (BV, SL, ST) in green, samples from *P. dactylifera* C242 (TU C242, DJ C242) in orange and *P. dactylifera* C254 (DJ C254, TU C254) in blue. Samples C242 from Djibouti show some admixture with *P. dactylifera* C254 group and Samples C254 from Tunisia show admixture with all groups.

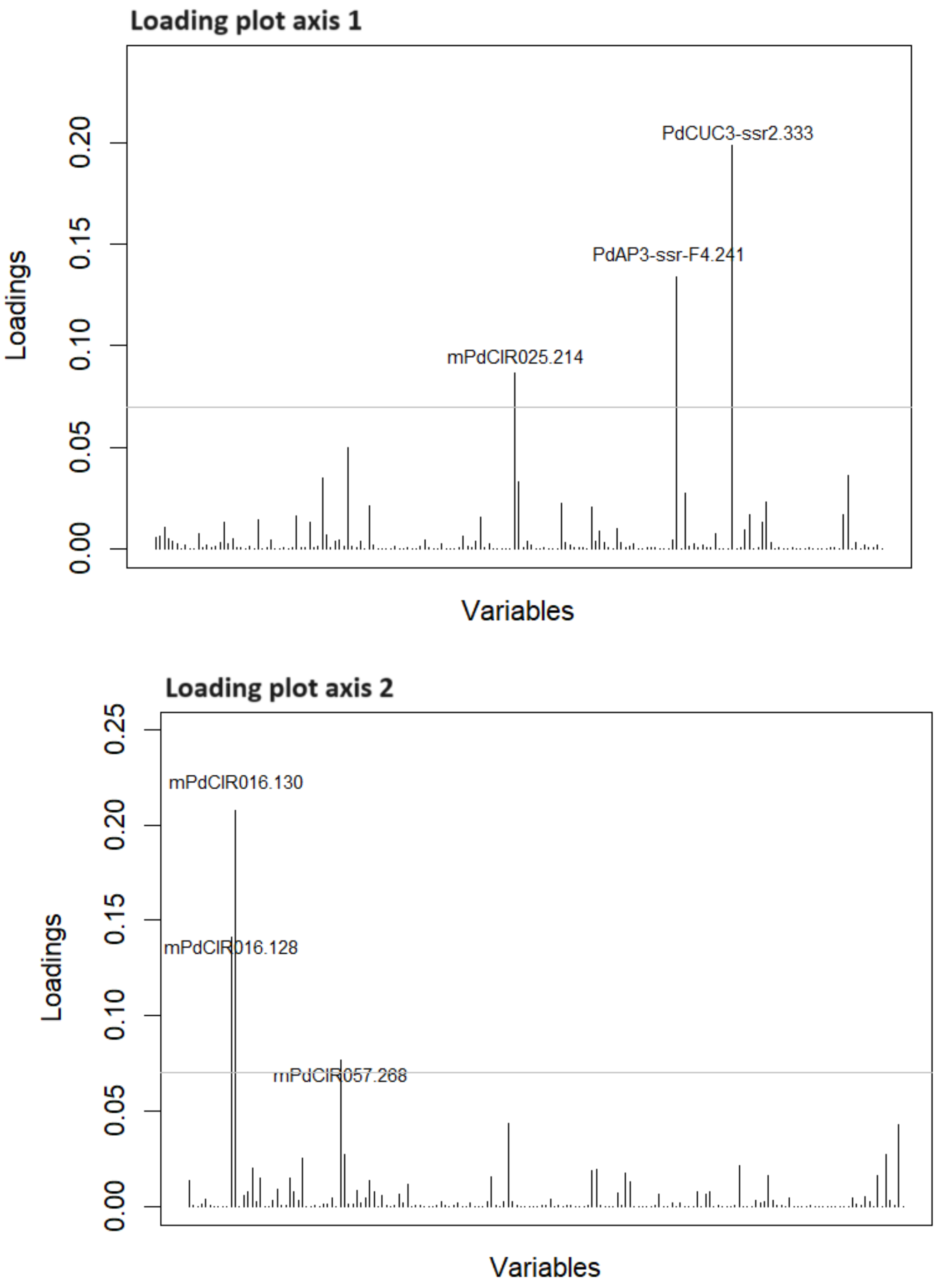

Figure S2. Loading plot for first (above) and second (below) axis in the Discriminant Principal Component Analysis (DPCA).

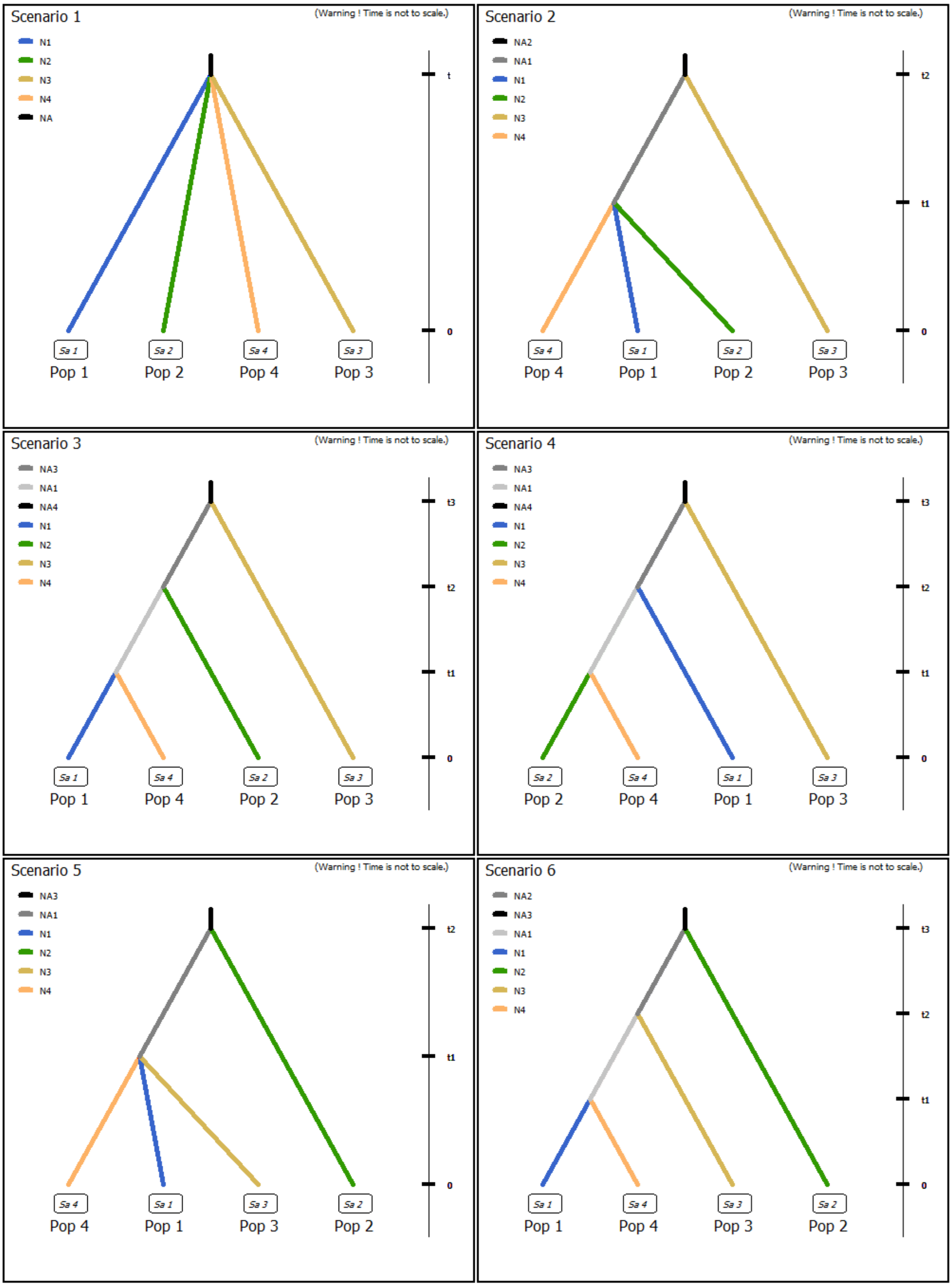

Figure S3. Six scenarios of population demography of *P. atlantica*, *P. dactylifera* and *P. canariensis* examined by ABC analysis as implemented in DIYABC v2.0 (Cornuet et al., 2014): a common split for all populations (scenario #1); ancestral divergence of two genetic pools (*P. atlantica* and *P. dactylifera* vs *P. canariensis*) and subsequent split into

different populations of *P. dactylifera* and *P. atlantica* (scenario #2); sequential split of *P. canariensis*, *P. atlantica* and simultaneous divergence of *P. dactylifera* (C242 and C254) (scenario #3); sequential split of *P. canariensis*, *P. dactylifera* C254 and simultaneous divergence of *P. dactylifera* C242 and *P. atlantica*, following the 'progression rule' from eastern to western populations in *P. dactylifera* and *P. atlantica* (scenario #4); ancestral divergence of two genetic pools (*P. atlantica* s *P. dactylifera* and *P. canariensis*) and subsequent split into different populations of *P. dactylifera* and *P. canariensis* (scenario #5); and, lastly sequential split of *P. atlantica*, *P. canariensis* and simultaneous divergence of *P. dactylifera* (C242 and C254). The samples were considered in the scenarios as each sampled population: Pop 1: *P. atlantica* from Cape Verde; Pop 2; *P. dactylifera* C254 from Djibouti, Pop 3: *P. canariensis* and Pop 4: *P. dactylifera* C242 from Tunisia. Ti time scale is measured in generations, where i ranged from 1 to 4; Ni effective population size of actual populations, where i ranged from 1 to 5; Nai effective population size of non-sampled ancestral populations, where i ranged from 1 to 4.

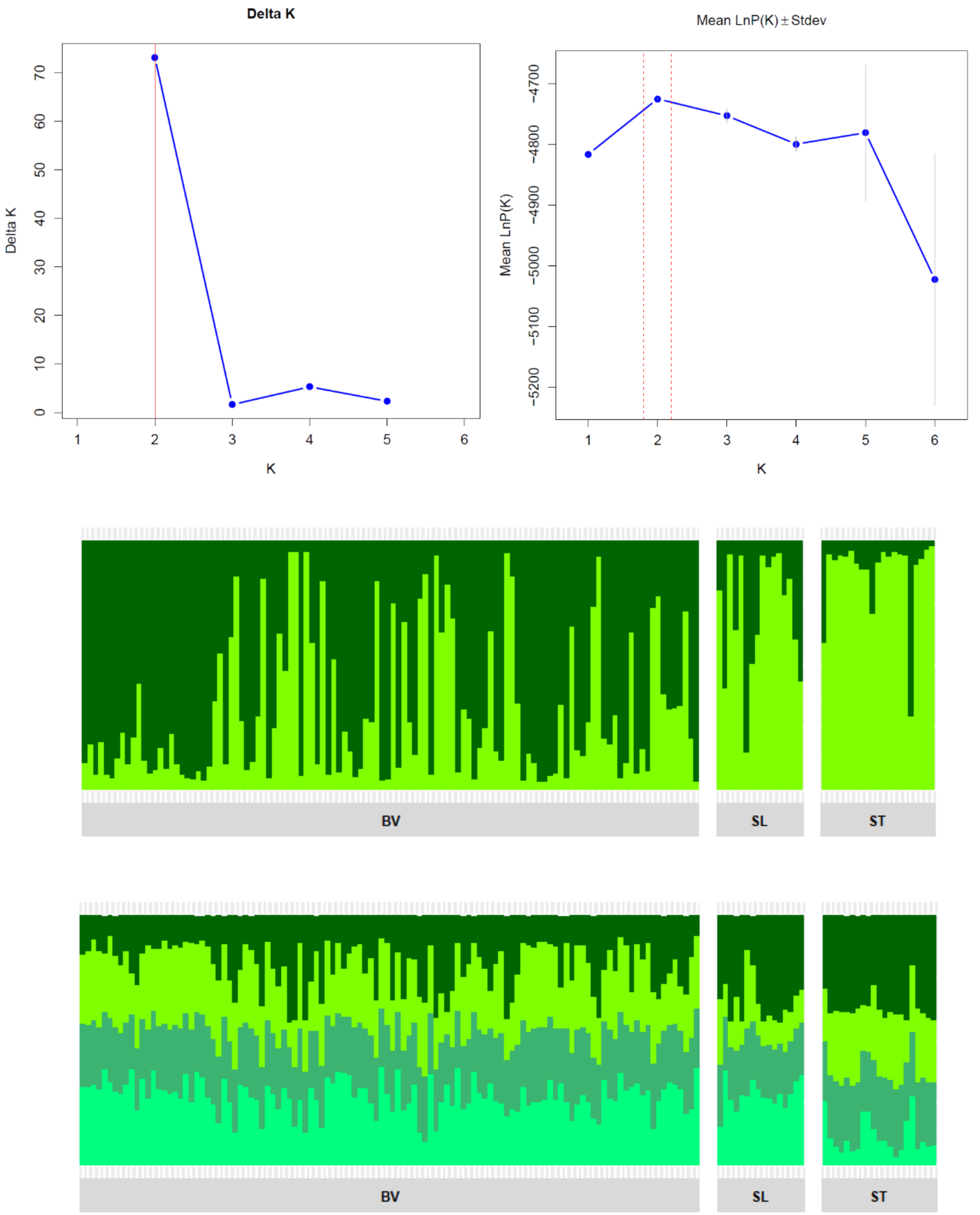

Figure S4. DeltaK and Mean L(K) Probability from STRUCTURE Analysis and Clustering Analysis Results. **Above**: The results indicate that K=2 is the best fit for *P. atlantica*, with K=4 as the next best fit. **Below**: The results of the structure analysis. At K=2, there is genetic structure between BV, SL, and ST. However, at K=4, no clear pattern emerges, and the genetic composition of individuals appears mixed.
